# Drivers of bird-window collisions in southern South America: a two-scale assessment applying citizen science

**DOI:** 10.1038/s41598-019-54351-3

**Published:** 2019-12-03

**Authors:** Natalia Rebolo-Ifrán, Agustina di Virgilio, Sergio A. Lambertucci

**Affiliations:** Grupo de Investigaciones en Biología de la Conservación, Laboratorio Ecotono, INIBIOMA (Universidad Nacional del Comahue – CONICET), Bariloche, Argentina

**Keywords:** Biodiversity, Urban ecology

## Abstract

Bird-window collisions are one of the main causes of avian mortality worldwide, with estimations reaching up to almost one billion of dead individuals annually due to this cause in Canada and the USA alone. Although this is a growing conservation problem, most of the studies come from North America, evidencing the lack of knowledge and concern in countries with high biodiversity and growing population development. Our objectives were: (1) to estimate the current situation of bird-window collisions in Argentina, a country with around 10% of the world’s avian biodiversity, and, (2) to identify drivers of bird-window collisions at a national and local scale, focusing on a city surrounded by a protected area. We used a citizen science project called “Bird-Window Collisions in Argentina” that consisted of an online survey that collected data on collision metrics and risk factors. We found that more than half of participants reported at least one collision during the last year, suggesting this issue is common and widespread. In addition, our data show that the number of windows and the presence of vegetation reflected in windows are factors that strongly influence the risk of collision at national scale. On the other hand, the environment surrounding buildings affects the rate of bird-window collisions at local scale, being greater in buildings surrounded by tall vegetation than in buildings surrounded by a greater proportion of urbanization (human-made structures). We call for attention on a topic that has been poorly evaluated in South America. We also encourage future scientific studies to evaluate additional risk factors and mitigation strategies accordingly, to provide a better understanding of bird-window collisions particularly in a highly biodiverse region as South America.

## Introduction

Modification of natural environments into urbanized areas following human population growth has well known negative impacts on wildlife^1–4^. Increased anthropogenic infrastructure can be a major cause of disturbance. In this sense, flying wildlife face increasing conflict in the airspace due to overlapping with man-made structures^5–8^. For instance, between multiple barriers birds are exposed to, windows pose one of the main threats, causing a great avian mortality^9–11^. Estimates of bird mortality from window collisions range from 16 to 42 million individuals annually for Canada, and from 365 million to nearly one billion for the United States^11,12^. Although this has been recognized since decades, this conservation threat for flying animals is increasing^9,13,14^. However, despite their potential impact on bird populations, bird-window collisions have been mainly studied in the Northern Hemisphere, revealing the lack of scientific attention to this issue in other regions of the world^15^.

Birds collision with man-made objects is a significant cause of avian mortality worldwide^6,8^, making essential to understand the causes that promote it in order to manage effective mitigation measures. Bird collisions occur when those animals do not recognize man-made objects as barriers, due to their visual and perceptual limitations^16^. Birds in flight may not perceive objects in the direction of flight because they usually direct their vision downwards or sideways, in search of food, predators or conspecifics^16^. Complementarily, the binocular vision of birds may not be high resolution, so even if birds direct their vision in the direction of flight, they may not perceive a static object^16^. Regarding bird-window collisions, several studies suggest that environmental resources, such as food and nesting sources, as well as the characteristics of the surrounding landscape and the structure of buildings may work as drivers of collisions^15^. Complementarily, glass may behave as a reflective or transparent material that causes the erroneous perception that the environment continues. A higher frequency of collisions has been observed in buildings with windows where adjacent vegetation was reflected^17–20^. Surrounding food sources, whether vegetation or artificial bird feeders, have also shown to increase collisions^21–23^. Moreover, configuration of the surrounding land cover of the building plays a fundamental role as a predictor of collisions, with the most urbanized environments, or with a higher level of development, having the lowest frequency of collisions^18,24–26^.

Although it is a worldwide threat, bird-window collisions has been studied mainly in North America, but poorly or non-studied in other parts of the world^15^. Importantly, many of the less studied regions include the countries with the greatest diversity of birds^27^. Fortunately, although it is still limited, there is a growing interest in studying this threat in Latin American countries. Some studies exist for Brazil^28,29^, Colombia^30–32^, Costa Rica^33–35^ and Mexico^36–38^. However, there is rare or nonexistent data for other large countries with high bird diversity, such as Argentina, were around 10% of the world’s bird diversity inhabit^39,40^.

Protected areas are designated for the protection and maintenance of biological diversity, however studies of bird-window collisions related to these areas are almost null (but see Santos *et al*. 2017). Considering that avian diversity is generally greater in natural than in urban areas^41^, the impact of bird-window collision near or within protected natural areas deserves special attention. This is particularly relevant for Argentina, since, although most of the national territory belongs to natural landscapes, only 8% are protected areas (https://www.protectedplanet.net/country/AR) and these areas are surrounded by human settlements with different degrees of urbanization or potential human influence^42^.

Here, we developed a citizen science project to evaluate bird-window collisions through a two-scale approach: (1) assessing the current situation of bird-window collisions in Argentina by comparing some collision metrics with those reported by previous studies using similar data collection procedures, and (2) identifying drivers of bird-window collisions at national scale and particularly at local scale in a city surrounded by a protected area in Argentine Patagonia. We predicted that buildings with more windows, with vegetation reflected in windows, and with bird attractors, such as feeders or vegetation that provides food, will present a greater collision risk. We also predict that vegetation composition from buildings’ surrounding will have an effect on the collision risk, with buildings located in more developed areas (i.e., where the proportion of pavement and human-made structures is greater than the vegetated area) being less risky.

## Methods

### Data collection

We assessed bird-window collisions regionally in Argentina, and locally in the city of Bariloche which is immersed in the Nahuel Huapi National Park, Patagonia (41°00′ S 71°30′ W, Río Negro), using a citizen science project called “Bird-Window Collisions in Argentina”. The study began on November 23, 2017, with an online survey distributed via different media including newspaper, email and social webs (Facebook, Twitter, and WhatsApp). We specifically sent e-mails with the survey link to (1) universities where degrees related to biological sciences are offered, (2) public research centers in biological sciences, and (3) the non-governmental organization (NGO) Aves Argentinas/AOP (https://www.avesargentinas.org.ar/). We asked these organizations to forward the survey link to their students, staff or associates. Complementarily, we published the survey on the Facebook webpage of our research group (https://www.facebook.com/GrInBiC/), and we asked for help to share it in order to increase the number of participants. Finally, we published an article in the regional newspaper “Rio Negro” from North-Western Patagonia to increase the number of participants at the local scale. (https://www.rionegro.com.ar/bariloche/investigan-la-muerte-de-aves-por-colisiones-contra-las-ventanas-BF4546120). Importantly, we asked every person to respond, no matter if they knew the problem or have seen any collision ever. Participants’ responses were optionally anonymous. Participants have been able to choose the possibility of providing their e-mails, which have been voluntarily requested for a possible extension of the study in the future. The principal researcher of the study was the only with access to the data provided by the participants, which were used exclusively for research purposes. This data has been the only identifying information of the participants and information that could reveal the identity of the participants will never be made public. A brief summary of the objectives of the study was provided and they were then asked to give their consent to participate in the research. Responses from participants that decided not to participate in the study (did not specifically accept to be part) but still answered all the questions, and from participants under the age of 18 were not included in the study.

The survey was available for one year. Participants provided data on the location and characteristics of their buildings and the surrounding environment. They also reported on the existence, if any, and number of collisions they remembered up to a year ago, employing a memory recall survey. This method is frequently used in citizen science studies^23^, and allows us to compare our data with previous studies^26^. Responses were then transformed into numerical or categorical variables to analyze potential drivers of collisions (see Table 1).

**Table 1:** Questions made to the participants from the online survey and resulting variables.

Question	Variable	Type of variable	Levels
How long have you lived in your building?	Months	Numerical	—
How long do you stay at home during daylight hours?	Hours	Categorical	More than 4 hours/Less than 4 hours
How many windows does your building have?	Windows	Numerical	—
Are there trees or vegetation that reflect on the windows of your building?	Reflection	Categorical	Yes/No
Are there trees or vegetation that provide food or nesting places for birds outside your building?	Natural_Attractor	Categorical	Yes/No
Do you have any artificial bird attractor outside your building? (feeders, nests, water fountains)	Artificial_Attractor	Categorical	Yes/No
Have you ever seen or heard a bird collide with the windows of your building?	Collision_Ever	Categorical	Yes/No
Have you seen or heard a bird collide with windows of your building in the past year?	Collision_Year	Categorical	Yes/No
How many bird collisions against your building windows do you remember until a year ago?	Collision_Number	Numerical	—
How many birds that collided on those occasions died?	Fatality_Number	Numerical	—
Which of these groups of birds collides most frequently with the windows of your building?	Group_Birds	Categorical	Raptor/Passerines/Pigeons/Hummingbirds//other bird species
What type of building do you live in?	Type_Building	Categorical	1 to 3 story-building/>3 story-building

The type of variable and the levels of each categorical variable are reported. The variables were included in the models or used in metric comparisons.

The variable Group_Birds was classified following previous studies suggesting collision differences among them^11,43,44^. For this purpose, we included silhouettes of the birds in the online survey in order to facilitate their identification to people with little experience in bird observation.

### Bird-window collisions in Argentina

To assess the current situation of bird collisions in Argentina, we compared the variables: Collision_Ever, Collision_Year, Collision_Number, and Fatality_Number, with the same collision metrics reported by Kummer *et al*.^26^. Both sets of variables were measured using similar data collection procedures.

### Drivers of bird-window collisions at the national scale

To evaluate the building characteristics affecting the probability of bird-window collisions at a national scale, we used a weighted generalized linear model (GLM) with a binomial distribution and logit link function, fitted with R glm function^45^. The response variable Collision_Ever was binary (i.e., 1: if collisions were detected, 0: if no collisions were detected), therefore a binomial distribution was assumed (with parameter N = 1, representing a Bernoulli process). We included the following co-variables to explain the probability that a collision occurs: the number of windows present in the building (Windows), the presence/absence of vegetation reflected in the window (Reflection), food sources or nesting sites in the vicinity of the building (Natural_Attractor), and artificial bird attractors such as feeders, drinkers, or artificial nesting sites (Artificial_Attractor). The only quantitative variable (Windows) was centered and standardized, and the correlation between all explanatory variables was evaluated by estimating the Variance Inflation Factor with the vif function of the R car package^46^. We fitted a weighted regression to weigh the observations as a function of the time the participants spends at their buildings (Hours), which could strongly influence the probability of observing a collision. A value of weight = 1 was assigned to surveys where participants reported that they spend more than 4 hours within the daily light period in their home; and a value of weight = 0.5 if the participant spent less than 4 hours. Additionally, because participants had a variable building period (from 1 month to more than 12 months), Months variable was used as an offset variable in the model.

In addition, we evaluated which factors affected the number of collisions observed. The number of bird collisions observed up to one year before answering the survey (Collision_Number) was used as the response variable, with the same covariates, weights and offset as in the previous model. The only quantitative variable (Windows) was centered and standardized, and the correlation between the covariates was evaluated using the same procedure described above. Because the response variable was a count and the model residues showed over-dispersion, a negative binomial distribution was assumed. This model was adjusted with the glm.nb function of the MASS package^47^.

Considering that all the data used in the different models come from different provinces of the country, the spatial autocorrelation in the residues of each model was evaluated using the R acf function, but no spatial dependence was observed (see Figs. S1–S3 from Supplemenary Material).

In order to describe which groups of birds collided in highest proportion in surveyed buildings of the country, we compared the levels of the variable Group_Birds. Finally, we used the variable Type_Building to identify potential variations in the probability of collision according to the structure of the building. For this purpose, two categories of buildings were selected: (i) 1 to 3-story buildings (i.e. single-family houses), and (ii) buildings with more than 3 storeys (i.e. multi-family residential structures). Participants were asked to respond just one survey per residence. Thus, a survey was obtained for each low-rise building (in the case of buildings of less than 3 storeys) and a survey per apartment in the case of high-rise buildings (>3 storeys).

### Drivers of bird-window collisions at the local scale

For the local scale analyzes, we related the answers of participants who provided the address of their buildings to immediately surrounding land cover. For this, we characterized the surrounding environment of buildings from the largest urban area immersed in the Nahuel Huapi National Park, a protected area comprising more than 7000 km^2^. Urban area includes the city of San Carlos de Bariloche (41°0.9′ 71°18′ W) and its urban surroundings, which are located in the ecotone areas between the ecoregions of the Patagonian Steppe to the east, and the Patagonian Andean Forest to the west. This gradient, added to the continuous population growth, makes that buildings present different environmental condition with various vegetation types in spite of being close. In order to assess the effect of the immediately surrounding land cover, we measured the percentages of three habitat types (tall vegetation [trees and shrubs], short vegetation [grass] and urbanization [roadways and buildings]) within a buffer of 25 m radius from the building. This buffer is a small-scale vegetation composition representing immediately surrounding land cover of the building. Most of the single-family buildings in the city of Bariloche are located on lots of 400–1000 square meters of park, therefore estimating the surrounding land cover in an area of 25 meters provides information about the environment of the lot of property. To do this estimation, we created the circular polygon in the QGIS software, and we then used Google Earth satellite images to estimate the predominant vegetation in the circumference of each sampled building. We classified each surrounding of the building by the predominant cover type and fitted a glm with Negative Binomial distribution (due to model overdispersion) for the number of observed collisions as a function of cover type in the surrounding area. We used weights and offset variable as explained above. To contrast between the three types of land cover from the surrounding environment, we used the emmeans function from the emmeans R package^48^.

## Results

### Bird-window collisions in Argentina

**Figure 1:**
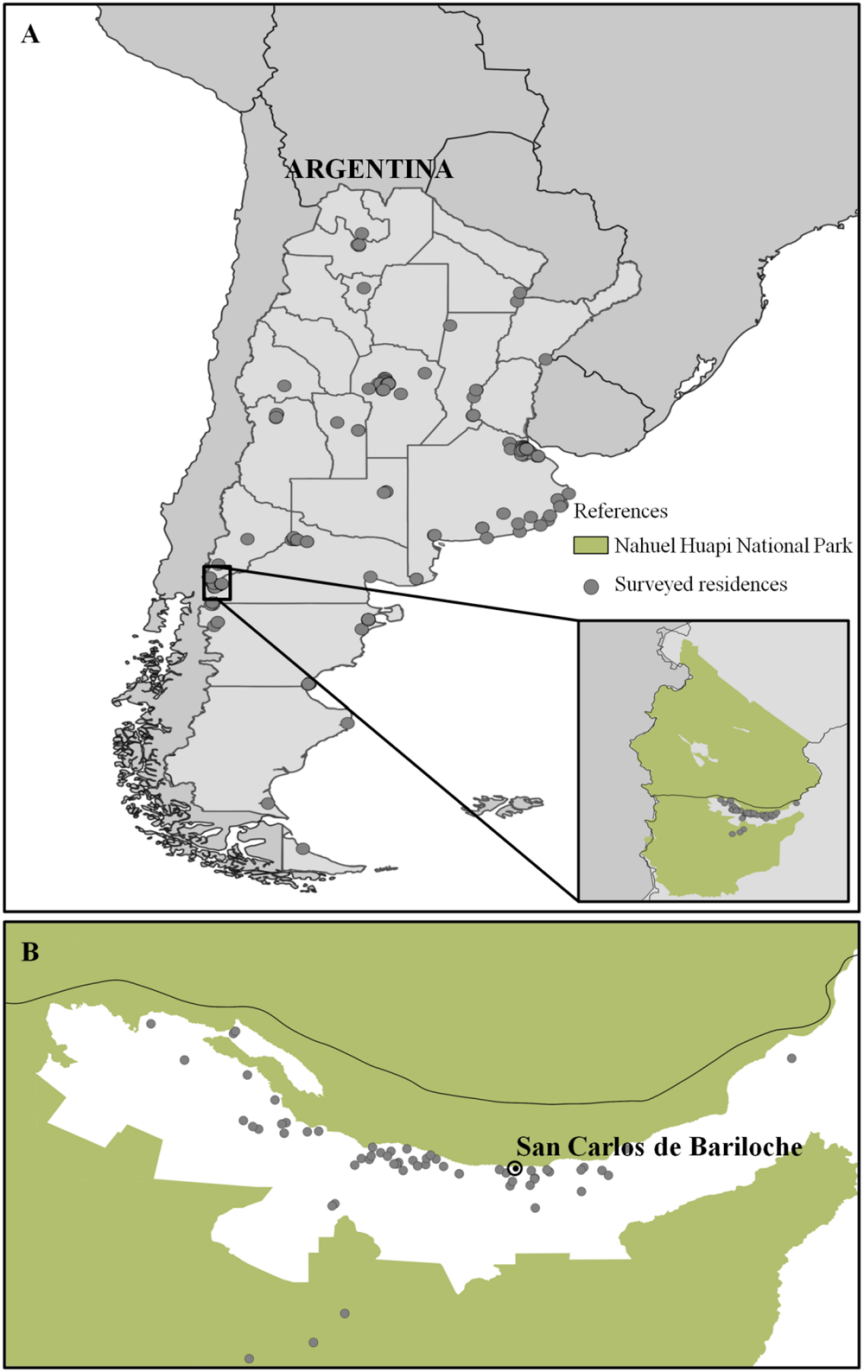
(**A**) Map of locations of surveyed buildings of Argentina (national scale study). (**B**) Detail of the exact location of surveyed buildings of the urban area surrounded by Nahuel Huapi National Park (local scale study).

We received a total of 356 complete surveys from 18 provinces of Argentina (Fig. 1). The 38.5% (137/356) of participants reported the precise address of their buildings. For the rest of the participants, the location of the buildings surveyed was placed with the highest degree of accuracy reported (province, city, neighborhood or street without number; in that order). From those participants who provided the information on how they received the survey (n = 337), 55.5% (n = 187) reported the survey was received via email, 36.8% (n = 124) via Facebook, 5.9% (n = 20) via a newspaper article, 1.2% (n = 4) via Twitter and 0.6% (n = 2) via WhatsApp.

Of the total number of participants that have lived in their building at least one year previous to the survey (n = 328), 52.7% (173/328) heard or saw a bird colliding with their building windows during the year. In addition, 75 of those participants (22.9%) reported that collisions resulted in the death of a bird, either instantaneously or subsequently, from collision injuries or predation by a domestic animal (cats or dogs). The number of annual collisions from memory recall survey was 3.7 ± 10.2 per building surveyed in Argentina, with a mean of 0.47 ± 1.22 fatalities registered annually per building.

We obtained collision metrics comparable and similar to those reported by Kummer *et al*.^26^, using similar and different techniques depending on the measured variable (Table 2). The probability of a bird colliding with a window (Collision_Ever) produced similar values to those studies previously reported, all rates slightly above 50% (Table 2). In our case, all participants who remembered a collision event at their building also remembered at least one collision for the year prior to the start of the survey (Collision_Year). Estimates of the number of collisions in a year (Collision_Number), and the number of collisions that resulted in avian fatality (Fatality_Number) were estimated using different methods than Kummer *et al*.^26^, however, we obtained similar results.

**Table 2:** Studies reporting metric measures of bird-window collisions.

	Kummer *et al*.^26^	Bayne *et al*.^21^	Our study
Participants	381	1458	328
Collision_Ever	56.5%	50.5%	52.7%
Collision_Year	43.9%	39.0%	52.7%
Collision_Number	5.55^*^	1.7	3.7
Fatality_Number	0.48^*^	0.7	0.47

Collision_Ever: the probability that a bird has collided with a window at some point in the past; Collision_Year: the probability that a bird has collided with a window in a year; Collision_Number: average of number of birds collided in a year; and Fatality_Number: average of number of collisions that resulted in a fatality in one year.

^*^Predicted values obtained by standardized carcass search data.

**Figure 2:**
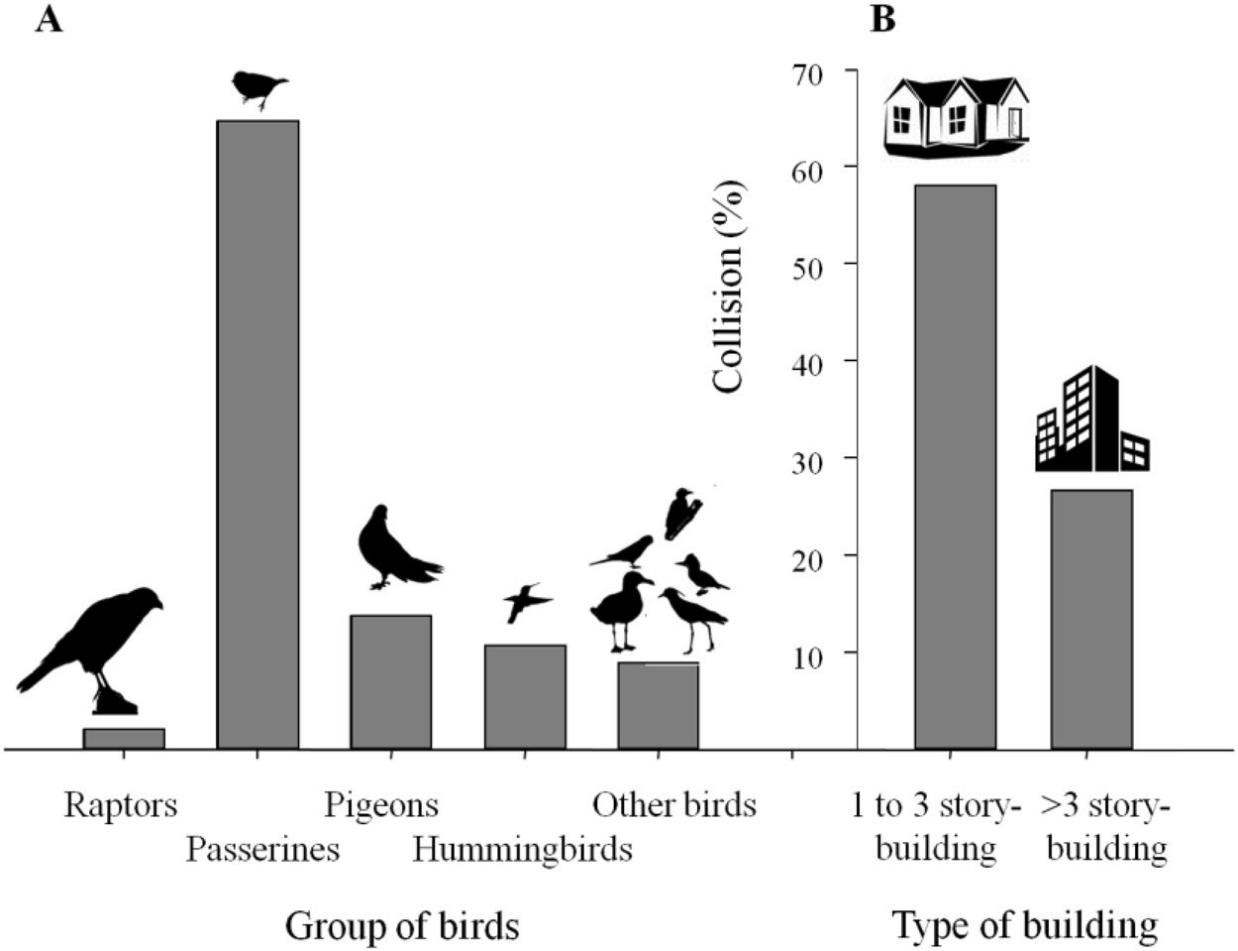
(**A**) Proportion of each group of birds (Group_Birds) that collide most frequently in the surveyed buildings of Argentina and; (**B**) Proportion of the two different types of buildings (Type_Building) with bird-window collision events based on the responses from the online survey.

Regarding the group of birds that collide with windows (Group_Birds), passerines were by far the most important collision group (64.6%, Fig. 2A). Pigeons, hummingbirds, and other bird species collided at rates similar to and below 15% each, while raptors were the group with the lowest collision rate (2.1%; Fig. 2A). Collisions differed in terms of the type of building surveyed, being higher in low-rise buildings (1 to 3 story-building) than in buildings with more than three stories (Fig. 2B). From the participants living in 1 to 3 story-building (n = 281), 58% responded that they heard or saw a collision at some point in the past, while for taller buildings (>3 story-buildings)s the percentage dropped to less than half (n = 75, 26.7%, Fig. 2B).

### Bird window-collision factors

#### National scale

The presence of vegetation reflected in the window increased the probability of bird-window collisions significantly. The base collision probability for buildings without reflected vegetation, natural or artificial bird attractors was 0.78 (Table 3), and it increased to 0.89 when there was vegetation reflected in the windows. Moreover, the probability of collision increased significantly with the number of windows (Table 3, Fig. S1 in the Supplemenary Material). For example, the probability for a building with 5 windows was 0.36, for a building with 10 windows it was 0.68, while the probability for a building with more than 15 windows was greater than 0.90 (Fig. S4, Supplementary Material). Finally, we did not detect any significant effect of the presence of natural or artificial attractors on the probability of collision (Table 3).

**Table 3:** Results of the model that evaluates the probability of collisions in relation to different variables at the national scale.

Coefficients	Estimates	Std. Error	z-value	95% Conf. Int.		p-value [Pr (>|z|)]
				Lower	Upper	
α_0_ (Intercept)	1.19	0.42	2.83	0.33	1.99	**0**.**005**
α_1_ (Reflection: Yes)	0.95	0.31	3.11	0.35	1.56	**0**.**002**
α_2_ (Natural_Attractor: Yes)	0.72	0.42	1.70	−0.09	1.59	0.089
α_3_ (Artificial_Attractor: Yes)	0.08	0.29	0.28	−0.49	0.65	0.780
α_4_ (Windows)	2.88	0.48	5.84	1.98	3.85	<**0**.**001**

We present the estimated value (Logit scale), standard error, z-value and their associated p-value, and the lower and upper limits of the 95% confidence interval. Significant p-values are highlighted in bold. α_0_ is the baseline probability representing the situation of buildings with no reflection and no natural or artificial bird attractors.

α_1_ is the regression coefficient that represents the effect of window reflection on the probability of collision; α_2_ is the regression coefficient that represents the effect of the presence of natural attractors surrounding the building; α_3_ is the regression coefficient that represents the effect of the presence of artificial attractors, and α_4_ is the regression coefficient that represents the effect of the number of windows on the probability of collision.

The estimated number of collisions in absence of vegetation reflected, natural or artificial attractors was 0.44 (Table 4). The rate of collisions observed per year increase with the presence of window reflection from 0.44 to 0.98 (Table 4). We did not detect statistically significant effects of the presence of natural or artificial attractors, nor the number of windows that the building has on the total number of bird-window collisions throughout the year (Table 4).

**Table 4:** Results of the model that evaluates the number of collisions in relation to different variables at the national scale.

Coefficients	Estimates	Std. Error	z-value	95% Conf. Int.		p-value [Pr(>|z|)]
				Lower	Upper	
β_0_ (Intercept)	−0.22	1.47	−0.15	−3.10	2.67	0.882
β_1_ (Reflection: Yes)	4.43	1.27	3.49	1.95	6.91	**<0**.**001**
β_2_ (Natural_Attractor: Yes)	−1.41	1.64	−0.86	−4.62	1.80	0.390
β_3_ (Artificial_Attractor: Yes)	0.61	0.52	1.17	−0.41	1.63	0.244
β_4_ (Windows)	−0.48	1.18	−0.40	−2.80	1.85	0.688

We show the estimated value (natural logarithm scale) of the intercept and regression coefficient of each variable, the standard error, the z-value and its associated p-value, and the lower and upper limits of the 95% confidence interval. Significant p-values are highlighted in bold. β_0_ is the baseline level of bird-window collisions representing the situation of buildings with no reflection and no natural or artificial attractors.

β_1_ is the regression coefficient that represents the effect of window reflection on the number of birds colliding; β_2_ is the regression coefficient that represents the effect of the presence of natural attractors surrounding the building; β_3_ is the regression coefficient that represents the effect of the presence of artificial attractors, and β_4_ is the regression coefficient that represents the effect of the number of windows on the number of birds that collide.

#### Local scale

**Figure 3:**
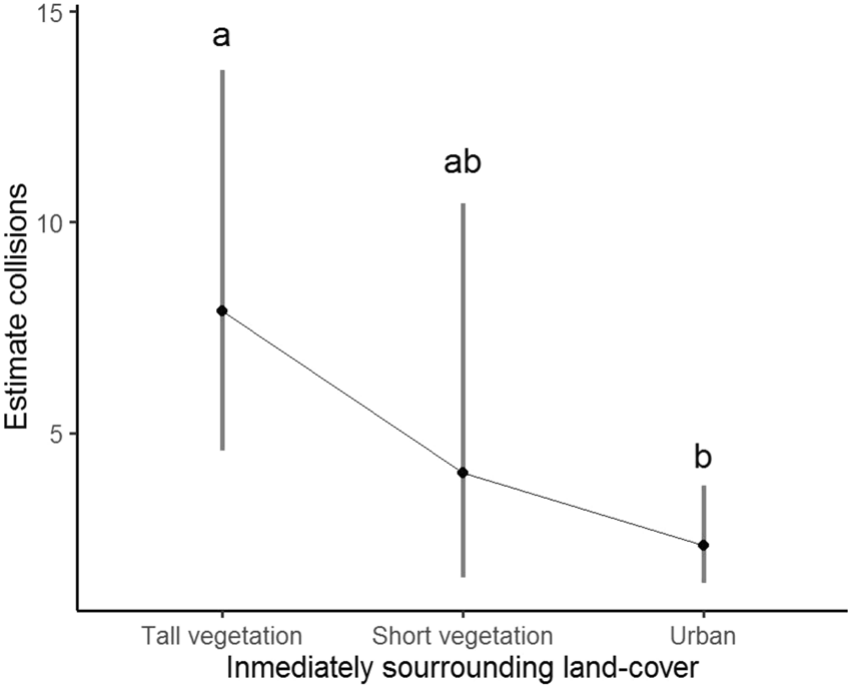
Estimated number of collisions per year for each type of environment surrounding buildings in urban areas immersed in the Nahuel Huapi National Park. Different letters indicate significant differences.

We received 61 surveys with the precise location of buildings from urban areas surrounded by a protected area in Patagonia (Fig. 1). We found that buildings that are immersed in a land cover matrix where the tall vegetation predominates, the number of collisions is significantly higher than in buildings where there is a predominance of urban areas (Table 5, Fig. 3). Buildings located in areas with short vegetation predominantly had an intermediate number of collisions between tall vegetation and urban areas (Fig. 3). We found a consistent pattern where the number of collisions decreases with the degree of urbanization (Table 5).

**Table 5:** Results for the model that evaluates the number of collisions as a function of the characteristics of the immediately surrounding land cover the buildings.

Coefficients	Estimates	Std. Error	z-value	95% Conf. Int.		p-value [Pr(>|z|)]
				Lower	Upper	
γ_0_ (Tall vegetation)	−0.32	0.28	−1.64	1.84	2.19	0.244
γ_1_ (Short vegetation)	−0.67	0.56	−1.20	−0.94	−0.13	0.231
γ_2_ (Urban)	−1.22	0.37	−3.30	−1.30	−0.71	<**0**.**001**

The estimated values (natural logarithm scale) of the intercept and regression coefficient of each variable, the standard error, the z-value, and its associated p-value, and the lower and upper limits of the 95% confidence interval are shown. Significant p-values are highlighted in bold. γ_0_ is the coefficient representing the tall vegetation (trees and shrubs).

γ_1_ is the regression coefficient for environments with a higher proportion of short vegetation (grasses), and γ_2_ is the regression coefficient for urban environments (roadways and buildings).

## Discussion

More than half of participants surveyed in the national territory reported at least one episode of birds colliding with windows from their building; and almost a quarter of those collisions resulted in bird fatality. In view of these results, we suggest that bird-window collisions in Argentina deserve urgent attention. Considering drivers of collisions, our results showed that vegetation reflected in windows and the number of windows are factors that strongly influence the risk of collision on a national scale, while the immediately surrounding land cover of the building is a good predictor of collision rate at a local scale.

We found that windows reflecting vegetation increase the probability and rate of bird-window collisions in the surveyed buildings in Argentina. This is consistent with previous studies which show that reflective windows represent a strong collision risk factor^16,26,49,50^, becoming up to three times more riskier than those that do not reflect vegetation^26^. In addition, our results showed that the number of windows per building was the most important factor in predicting the probability of bird-window collisions, which results in a building with more than 15 windows having a more than 90% probability of a bird colliding if no mitigation measures are taken (Fig. S1 in the Supplemenary Material). However, we did not find support for an increasing frequency of collisions as the number of windows increased. The lack of association between the number of windows and the number of collisions in this study may be due to the fact that the windows of a building may present differential collision risks due to various factors such as the structure and position of the windows^9,49,51^, and also the surrounding environment^52^. Birds often collide with particular windows in a building^52^, therefore, a larger number of windows in a building would not necessarily imply an increase in the frecuency of collision. However, the probability of a bird colliding at some point will be increased if the area of glass increase.

We did not find a consistent pattern of an increased collision risk due to the presence of natural or artificial bird attractors. This negative result could be due to the fluctuation of natural food with the season of the year. Southern South America is characterized by a marked climatic seasonality in which natural foods fluctuate between seasons. We did not consider this potential effect in our study, so any positive association between artificial bird attractors and risk of collision in seasons where food is scarce could be diluted by analyzing all seasons together. In addition to this, despite bird attractors have been suggested as factors of risk for collision as a consequence of increasing bird abundance, most studies are exclusively on bird feeders^18,19,22,24^. In this study we include water fountains, artificial nest sites and bird feeders as artificial bird attractors, which could vary in the abundance of attracted birds. This may explain the lack of positive association between artificial bird attractors and the risk of collision. Finally, we should note that in Argentina the bird feeders most used are those for hummingbirds, and this may also influence our results.

Environmental factors at local scale as the land cover matrix adjacent to the surveyed buildings have a marked effect on the annual frequency of collisions. Our results agree with previous studies that found that green areas surrounding building influence the collision risk, being collisions less frequent in highly urbanized environments and more frequent in buildings surrounded by green areas^18,25,30^. The fact that taller vegetation increase collision risk is consistent with the results on a national scale, which indicated that windows reflecting vegetation are the factor most strongly associated with increased bird-window collisions. In this sense, Bariloche city, is composed of areas that differ in their degree of urbanization. While the small city center is characterized by a greater amount of human constructions, such as pavement and low-rise building, the rest of the city has houses immersed in a matrix of vegetation similar to that of the Nahuel Huapi National Park, that surround the city. This could be leading to an ecological trap, where the birds would be selecting the potentially better areas within the city because they are least urbanized, with the consequent exposure to this anthropogenic threat.

Overall, our findings on collision metrics in Argentina were similar to those reported for Canada (Table 2). Still, some differences were found that may be due to the different data collection methods used. The number of birds collided in a year (Collision_Number) showed here, as well as Bayne *et al*.^21^ results, were estimated by memory recall surveys. In both cases, this variable showed lower values than those reported by Kummer *et al*.^26^, who used the standardized carcass search method. Although the memory recall surveys provides a quick, inexpensive, and passive way to obtain data, making it a highly effective method, the results obtained could be underestimated, because people may have not remembered some or many of the collisions and they may have reported only collisions from recent weeks or months. However, the result on bird mortality over a year (Fatality_Number) in our study was closer to the value reported by Kummer *et al*.^26^, with standardized carcass search method, than to the value reported by Bayne *et al*.^21^, who used the same method of data collection as this study. Although it is likely that people tend to remember more easily the collisions that resulted in a fatality, leading in results closer to the real - or obtained by standardized carcass search method - we encourage future studies to collect real collision data in our study area, in order to make more reliable comparisons. Therefore, even though the estimations of this methodology should be taken with caution, our data may be used for future comparisons with the same or similar data collection techniques, as we did here.

Participants reported 50% more passerine collisions than other groups of birds. These results are consistent with previous studies both in the Northern Hemisphere^11,33,50^, and in South America, where, as we also observed in our study, passerines and pigeons were the most frequent victims^28,29^. While collisions have been explained by the relative abundance of each taxa, there are other characteristics that may influence the differential risk of collision, such as age, sex, behaviour or migratory capacity^12,14,16,18^. In this sense, it has been proposed that birds with a small wing area and rapid flight, such as passerines may be more likely to collide with windows because they are less able to react quickly to unexpected obstacles^53^.Therefore, before analyzing other species-specific characteristics that might influence the risk of collision, it is necessary to conduct relative abundance studies to assess whether species collide according to their abundance or there are other species-specific traits^52^. However, susceptibility to death by collision may also differ between species and families, therefore the knowledge of which group of birds collide most frequently and which of them died provides key information thay will influence differently to the conservations of those species.

Participants living in 1 to 3 story-buildings reported more collisions than those living in buildings over 3 storeys. Although assessing the relative risk caused by single and multi-family buildings would require estimating the number of each type of building and extrapolating the number of collisions per apartment in high-rise buildings, our results, while providing descriptive data, agree with other studies that conclude that low-rise buildings contribute to the higher proportion of bird deaths caused by window collisions^11,12^. Furthermore, considering that the abundance of birds decreases with height^8,54^, and that houses or low-rise buildings are more likely to present vegetation that may be reflected in windows around them, we suggest that potential impact of buildings would be lower than that of houses. To test this, future researchs should consider how the vegetation reflected in the windows varies with the height of the building ans evaluate if there is a differential risk of collisions with this factor.

We obtained collision metrics similar to Canada, a country where about 25 million birds have been reported killed by this cause annually^12^. We cannot say that in Argentina the problem is of a similar magnitude as in Canada, since it would be necessary to estimate the number of buildings in the country, an objective not proposed in this article. However, we show that the collision of birds and windows in Argentina is, at least, an issue that deserves more attention due to its high, and until now unknown, collision metrics. More research is needed to know the actual magnitude of birds-window collisions in Argentina and thus estimate the potential effects on bird populations.

## Conclusions

Bird-window collision studies are scarce in Latin American countries and almost non-existent in protected areas. This work it the first estimates of avian fatality by window collision in southern South America, and although the values coul be underestimated, they are comparable to other studies. We showed that the number of windows and presence of vegetation reflected in windows are factors that strongly influence the risk of collision on a national scale, while buildings surrounded by tall trees represent a greater risk of collision for birds than those inserted in a predominantly urban matrix. This is extremely important and poses a greater problem if buildings are surrounded by protected areas, as is our study area. It is important to pay attention to an issue poorly known in South America and encourage future scientific studies to evaluate other risk factors, such as species-specific traits and seasonal and environmental factors to provide better insights for bird-window collisions. We also call to implement the mitigation strategies known for the Northern Hemisphere and validate their effectiveness for this area.

## Supplementary information

Supplementary material
