# Supplementary material for: Drivers of bird-window collisions in southern South America: a two-scale assessment applying citizen science
Source: Sci Rep. 2019 Dec 3;9:18148. doi: 10.1038/s41598-019-54351-3 (PMC6890675; doi:10.1038/s41598-019-54351-3)

# Supplementary material

**Drivers of bird-window collisions in southern South America: a two-scale assessment applying citizen science**

Natalia Rebolo-Ifrán^a^, Agustina di Virgilio^a^, Sergio A. Lambertucci^a^

^a^Grupo de Investigaciones en Biología de la Conservación, Laboratorio Ecotono, INIBIOMA (Universidad Nacional del Comahue – CONICET), Bariloche, Argentina.

Corresponding author: Natalia Rebolo

e-mail address: nataliarebolo@gmail.com; nataliarebolo@comahue-conicet.gob.ar

## Figure S1: Auto-Correlation Function (ACF) fitted to the residuals from the assessing the probability of collisions in relation to different variables at the national scale. The ACF function provides the autocorrelation values (y-axis) of a series with its lagged values (x-axis), and show if the current value depends consistently on previous values (the lags). The blue dashed lines represent the lag-wise 95% confidence intervals centered at zero. These intervals are used for determining the statistical significance of an autocorrelation estimate at a given lag versus a null value of zero, i.e., no autocorrelation at that lag. This means that, if a bar is above or below the dotted blue lines it means that a significant autocorrelation exist at that lag value. Recall that the ACF at lag-0 is always 1.


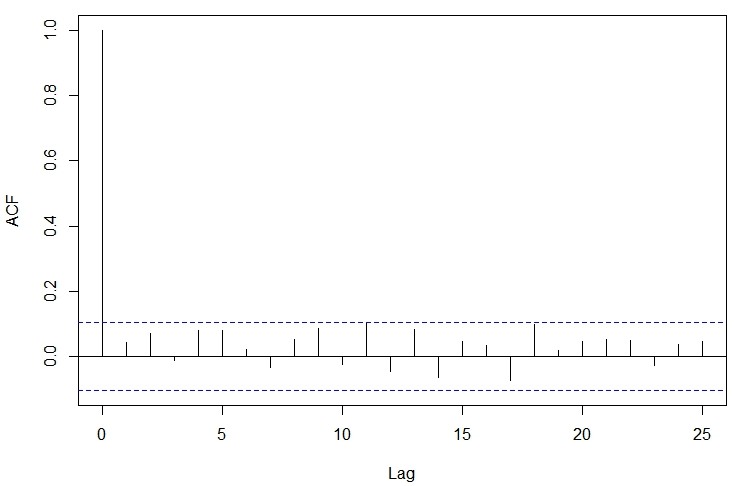


## Figure S2: Auto-Correlation Function (ACF) fitted to the residuals from the assessing the number of collisions in relation to different variables at the national scale. The ACF function provides the autocorrelation values (y-axis) of a series with its lagged values (x-axis), and show if the current value depends consistently on previous values (the lags). The blue dashed lines represent the lag-wise 95% confidence intervals centered at zero. These intervals are used for determining the statistical significance of an autocorrelation estimate at a given lag versus a null value of zero, i.e., no autocorrelation at that lag. This means that, if a bar is above or below the dotted blue lines it means that a significant autocorrelation exist at that lag value. Recall that the ACF at lag-0 is always 1.


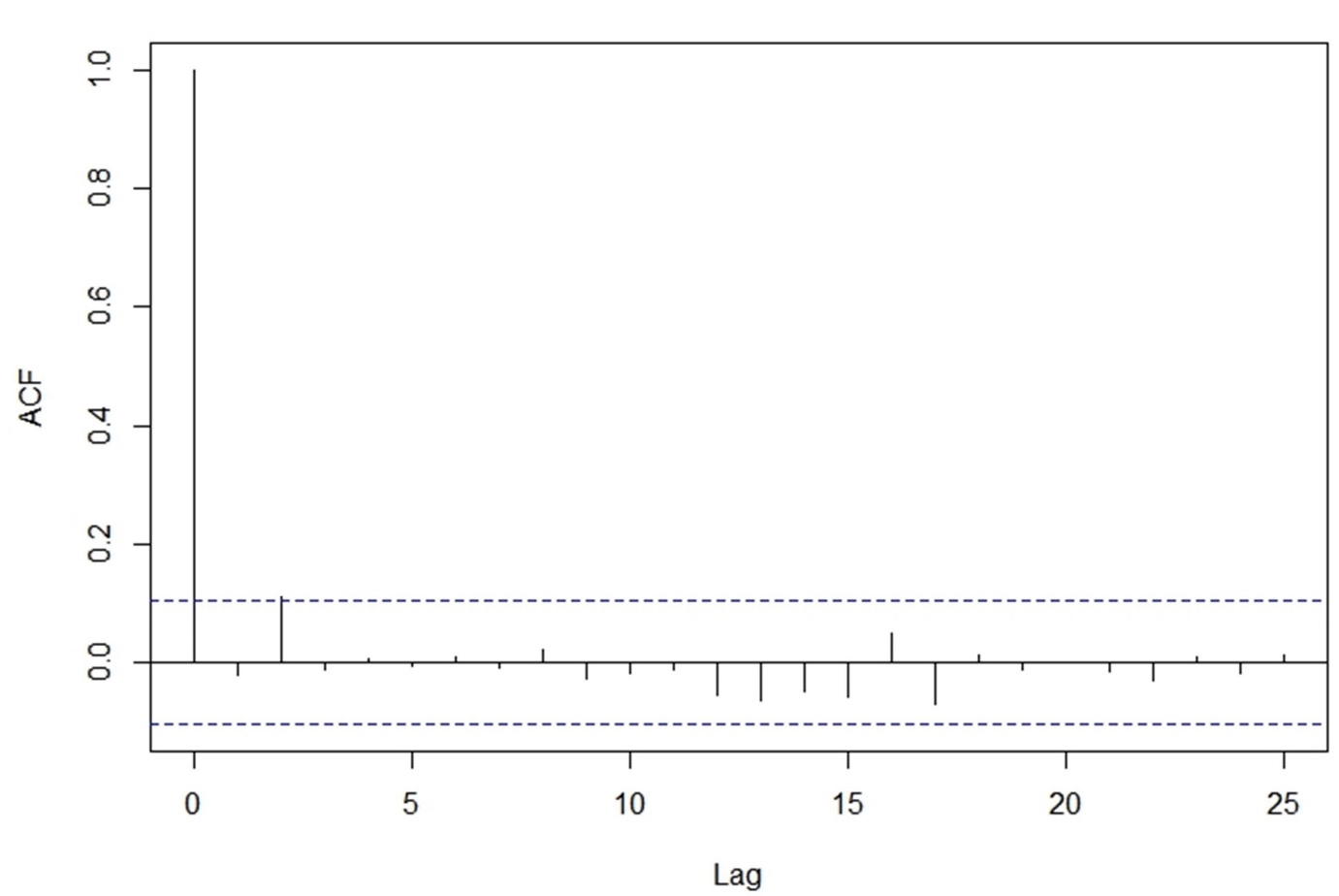


## Figure S3: Auto-Correlation Function (ACF) fitted to the residuals from the assessing the number of collisions as a function of the characteristics of the landscape around the buildings. The ACF function provides the autocorrelation values (y-axis) of a series with its lagged values (x-axis), and show if the current value depends consistently on previous values (the lags). The blue dashed lines represent the lag-wise 95% confidence intervals centered at zero. These intervals are used for determining the statistical significance of an autocorrelation estimate at a given lag versus a null value of zero, i.e., no autocorrelation at that lag. This means that, if a bar is above or below the dotted blue lines it means that a significant autocorrelation exist at that lag value. Recall that the ACF at lag-0 is always 1.


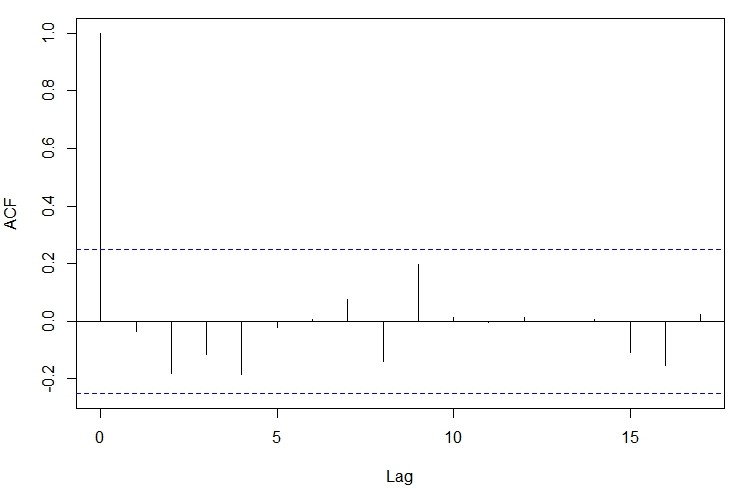


## Figure S4: Probability of bird-window collisions depending on the number of residence windows. Black dots are the observed collision values for each window number value. Red line represents the estimate of how the probability of collision increases as the number of windows increases.


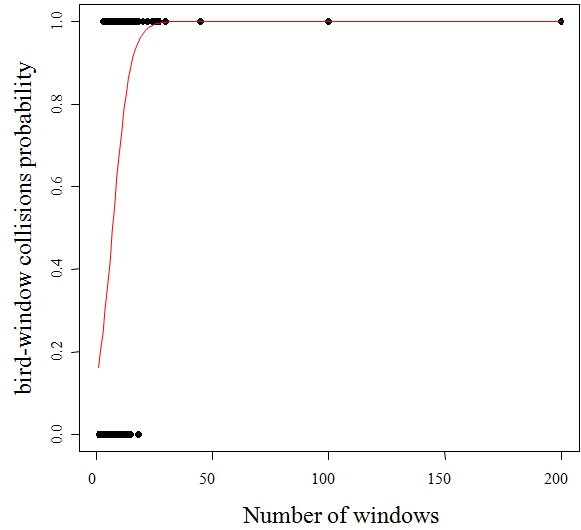

Supplement: Supplementary file 1 — Supplementary material [file 41598_2019_54351_MOESM1_ESM.docx]
